# Nutritional availability and carbon footprints of omnivorous, vegetarian and vegan diets: A cross-sectional analysis of dietary data for UK children aged 2–12

**DOI:** 10.1371/journal.pone.0342629

**Published:** 2026-06-18

**Authors:** Alice Coffey, Robert Lillywhite, Oyinlola Oyebode

**Affiliations:** 1 School of Cross Faculty Studies, University of Warwick, Coventry, United Kingdom; 2 School of Life Sciences, University of Warwick, Coventry, United Kingdom; 3 Wolfson Institute of Population Health, Queen Mary University of London, London, United Kingdom; Federal University of Minas Gerais: Universidade Federal de Minas Gerais, BRAZIL

## Abstract

As plant-based (PB) diets become more common among UK children, understanding their nutritional adequacy and environmental impact is vital. This study addresses that lack of understanding through assessment of the nutrient content and greenhouse gas emissions for omnivorous, vegetarian, and vegan diets. A cross-sectional analysis was conducted using three-day weighed food diaries from 39 UK children aged 2–12 years (omnivore n = 15; and PB: vegetarian n = 11; vegan n = 13). Nutrients were analysed with and without supplementation using Nutritics software. GHG emissions were calculated at the ingredient level (kgCO₂e/day) and grouped by Eatwell Guide food categories. No dietary group met all nutrient reference values. Omnivores exceeded recommended intakes for saturated fat and free sugars while failing to meet the recommended intake for fibre, whereas PB children had intakes of these nutrients in the healthy range. PB diets were adequate in protein and vitamin B12 even in the absence of supplementation. Vegan children also met iron requirements from diet alone, whereas omnivore and vegetarian children did not meet iron targets without supplementation. Vitamin D intake was insufficient across all groups when supplements were excluded, with only vegan children achieving recommended levels through supplementation. Zinc requirements were met only by vegetarian children with the aid of supplements and were not met by vegan or omnivore children with or without supplementation. Iodine intake remained inadequate in vegan children even with supplementation. Mean daily greenhouse gas (GHG) emissions differed significantly between diet groups, with omnivores having the highest emissions, while vegans had the lowest emissions: 46% lower than omnivores, and 20% lower than vegetarians. Well-planned PB diets can meet most nutrient needs in UK children when supported by fortified foods and supplements, while substantially reducing dietary GHG emissions compared with omnivorous diets. Shifting away from animal protein and dairy provides an opportunity for improving both nutritional quality and environmental sustainability.

## Introduction

Interest in plant-based (PB) diets has increased in recent years, with nearly half of UK residents reporting efforts to reduce their meat consumption [1]. Although many of these individuals still consume some animal products, this shift shows a move towards more vegetarian and vegan diets. Although there is no robust evidence of prevalence, an online poll completed by YouGov found that 2% of the UK population identified as vegan (diets containing no animal products) and 6% as vegetarian (diets containing no meat or fish); for those aged 18–24 this rose to 5% and 11%, respectively [2]. While a growing body of evidence supports the nutritional adequacy for more PB diets (used here as a combined term for vegetarian and vegan diets) in adults [3,4], there is limited evidence of their nutritional adequacy in children, particularly in the UK context.

As the adoption of PB diets increases, it is essential to understand if these diets can meet children’s nutritional needs. This is of particular importance in light of the “double burden of malnutrition” that many high-income countries, including the UK, are now facing in children, with rising obesity rates and increasing micronutrient deficiencies [5]. Data from the National Diet and Nutrition Survey (NDNS), shows children in the UK are at risk of deficiency in key nutrients, in particular, vitamin D, fibre, folate and iron, while exceeding recommendations for free sugars and saturated fat [6]. These findings reinforce the need to evaluate if PB diets can provide adequate nutrition for children without adverse effects.

Some studies have highlighted potential concerns with PB diets in children, a recent systematic review including 30 global studies with children aged 2–18, reported that children following PB diets may be at risk of deficiencies in iron, zinc and B12 [7]. Additionally, both PB children and omnivore children were at risk of deficiency of vitamin D, calcium and iodine [7]. However, a recent cross-sectional study undertaken in Germany, with 820 participants aged 1–18 showed that PB diets offer some health benefits and are higher in fibre and lower in saturated fat and free sugars, compared with omnivore diets [8]. Diets high in red and processed meats have been linked to increased risk of cardiovascular disease, certain cancers and type 2 diabetes (T2D) [9]. A Polish study found that children consuming vegan diets have healthier cardiovascular risk profiles but lower bone mineral content [10], but the long-term evidence remains unexplored.

Beyond nutritional considerations, the environmental impact of different dietary patterns is of increasing relevance. Food production contributes significantly to global greenhouse gas emissions (GHGEs), with animal-based foods, particularly red and processed meat, among the most emissions-intensive components of the food system [11,12]. Evidence from UK adults consistently shows that vegetarian and vegan diets are associated with substantially lower GHGEs than omnivore diets [13,14]. However, evidence on the carbon footprints of children’s diets remains limited, particularly in the UK context, where no studies have been conducted to date.

This study aims to address the critical gap in the literature by analysing the nutritional composition and carbon footprints of omnivore, vegetarian and vegan diets in UK children aged 2–12.

## Methodology

### Study design and participants

This study is a cross-sectional study of dietary data from UK children. Recruitment for this study began on 30/03/23 and ended on 15/11/23. Participants were recruited online via convenience and snowball sampling, with the electronic invitation circulated on social media. Inclusion criteria were those with omnivore, vegetarian or vegan children, between the ages of 2 and 12 years, living in the UK. Excluded participants were those who only had children outside the 2–12 age range in their care, or who lived outside the UK, those who followed a different diet to vegan, vegetarian or omnivore (e.g., pescatarian) and those with medical conditions that caused them to have a highly restrictive diet. This age range was chosen to align with UK Eatwell Guide, which apply from two years of age onwards [15]. The upper limit was chosen to capture primary school age children, representing a key developmental period, where dietary habits are established [16]. Parents participating with their children received a £10 shopping voucher for taking part, this amount was chosen to encourage participation, without being considered a bribe. Nutritional outcomes were also shared with parents. Research ethics approval was obtained from the University of Warwick’s Biomedical Sciences Research Ethics Committee (BSREC). Application reference: BSREC 76/22–23. Informed consent was obtained from all participating parents or guardians prior to data collection. This was done via electronic consent forms, sent and returned via email. In addition, children aged 10 and over provided assent using an age-appropriate information and assent form, which was also sent and returned via email.

### Sample size

The sample size for this study was restricted to 15–45 children based on available resources, including funding and time. There are no UK studies that compare vegan, vegetarian and omnivore diets in children; however, it is common for weighed dietary studies to have similar sample sizes to this to give indicative results [17–19].

### Nutrition assessment

Dietary intake was assessed using three-day weighed food diaries, completed by parents of the participating children through the Qualtrics survey software online. Electronic kitchen scales and instructions were provided for parents to weigh all food and drink that their child was served. Leftover food was also recorded, using fraction estimations – e.g., half the broccoli left. For school lunches, where parents could not weigh the food, meals were recorded and study staff estimated quantities using the school food plan portion guide as a reference. Parents also asked children if they left any food. Breast milk was recorded through minutes fed, with average amounts per minute used to estimate quantities. Parents were asked to provide brands of food, drinks and supplements consumed to allow for more accurate analysis. Where these weren’t provided, the most popular product from the Nutritics database were used [20], for plant milks, these were a fortified version. When brand information was not reported, the most used PB milk on the Nutritics database for that type of milk (soya, oat or almond) was used as a proxy. This database is based on UK and Ireland products. Dietary data were entered into Nutritics software [20] and nutrition reports were generated.

### Demographic data

Demographic data were collected online using Qualtrics. Demographic questions asked were:

- Relation to child participating
- Postcode (First part only, e.g., CV4)
- Household income bracket
- Qualifications
- Marital status

Some non-identifiable data about the participating children was also collected:

- Child’s sex
- Age
- Ethnicity
- School lunch status
- What type of school does the child go to (private, state etc.)
- What dietary pattern does the child follow

The demographic data collected helped identify trends and associations. Parents were asked the sex of their child, rather than gender, to compare with dietary reference values set for male and females.

### Data analysis

#### Demographic characteristics.

The demographic data collected were analysed by dietary pattern. This provided insights into the characteristics and distribution of the participants within each dietary group. Statistical analysis of the demographic variables was conducted using SPSS software [21]. Analysis of Variance (ANOVA) tests were performed to determine the significance of differences in characteristics between the groups with continuous data (age and index of multiple deprivation) and chi-squared used for categorical data (child ethnicity, school type, lunch type, child sex, parental qualifications, marital status, income group), with post-hoc tests conducted to see significant differences between dietary patterns.

#### Nutrients.

Dietary data were entered into Nutritics software (version 6.14) to calculate macro- and micronutrient intakes, including energy, total fat, saturated fat, protein, carbohydrate, fibre, free sugars, vitamins A, C, B12 and D, sodium, iodine, iron, calcium, zinc and folate. These nutrients were selected based on previous literature and UK government data identifying nutrients of concern in children [6,8,10]. These results were then entered into SPSS [21], where they underwent statistical analysis.

Mean nutrient intakes were calculated for each dietary group and compared using one-way ANOVA. Analyses were conducted both including and excluding dietary supplements to assess the impact of supplementation. Normality of nutrient data was assessed using the Shapiro–Wilk test; although some variables showed minor deviations from normality, group sizes were comparable and ANOVA was considered appropriate.

Nutrient intakes were compared with UK dietary reference values. For micronutrients, Reference Nutrient Intake (RNI) values were used; for energy, Estimated Average Requirement (EAR) values were applied; and for macronutrients, fibre and free sugars, age- and sex-specific UK recommendations were used. Intakes were expressed as a percentage of the relevant recommendation, and mean percentages were compared across dietary groups using ANOVA.

#### Carbon footprint analysis.

Based on the dietary data collected, detailed product and ingredient lists were generated for each dietary pattern, identifying exactly what was consumed by each participant. Carbon footprint values were estimated at ingredient level. Where available, values were sourced from established databases (primarily CarbonCloud and LiveLCA) [22,23], with preference given to UK-specific data or the closest geographical equivalent.

For ingredients lacking direct carbon data, estimates were derived using the closest comparable product, supported by expert judgement. For example, in the absence of a value for a vegan croissant, the footprint of a standard butter-based croissant was used, with a 50% reduction applied to account for the absence of dairy fat. Where no suitable proxy was available, carbon footprints were calculated from the ingredient composition. For instance, the carbon footprint of a ‘Yoyo Bear’ fruit snack (65.8% apple, 32.9% pear, 1% strawberry, 0.3% black carrot extract) was estimated using weighted averages for apples and pears.

All carbon values were standardised to kgCO₂e per 100 g to ensure comparability. These were then scaled to reflect the actual quantities consumed by each child, as recorded in the three-day weighed food diaries. This enabled the calculation of each participant’s daily dietary carbon footprint, from which mean values were derived across the study period.

Individual dietary data were then grouped into the Eatwell Guide food categories: fruit and vegetables; starchy foods/carbohydrates; protein (including meat, fish, pulses and PB alternatives); dairy (including dairy alternatives); fats (oils and spreads); and HFSS foods (crisps, biscuits, cakes, condiments). A further ‘other’ category was created for items not captured by the above (e.g., vinegar, spices). Composite foods were allocated according to their primary ingredient (e.g., margarita pizza to carbohydrate, pesto to oils and spreads).

Daily mean GHGEs were calculated for each individual across these categories. Overall group means were then compared using one-way ANOVA with post-hoc tests conducted in SPSS to assess differences between dietary patterns. Eatwell Guide categories were chosen to align the analysis with national dietary recommendations and to identify which food groups contribute most to dietary GHGEs in different dietary patterns, thereby supporting policy-relevant interpretation.

## Results

### Demographics

The demographic results are presented in Table 1, with additional variables reported in supplementary table 1 (S1 File). The study population was comprised of 39 participants: 15 omnivores, 11 vegetarians, and 13 vegans.

**Table 1:** Demographic and dietary characteristics of study participants included in quantitative dietary analysis (children aged 2–12 years).

Diet Pattern (overall % of participants)	Vegan (33%)	Vegetarian (28%)	Omnivore (39%)	*P-Value
**Study Size 39**	13	11	15	n/a
**Supplement use**				
**Yes**	12 (92%)	9 (82%)	2 (13%)	<0.001
**No**	1(8%)	2 (18%)	13 (87%)	
**Diet Pattern (overall % of participants)**	**Vegan (33%)**	**Vegetarian (28%)**	**Omnivore (39%)**	*P-Value
**Sex**				
**Female**	4 (31%)	4 (36%)	6 (40%)	0.878
**Male**	9 (69%)	7 (64%)	9 (60%)	
Age (years) **	Mean = 5.2 (std.dev = 2.68)	Mean = 5.73 (std.dev = 2.72)	Mean = 5.23 (std.dev = 2.80)	
**2-5**	7	5	9	0.870
**6-8**	5	4	3	
**9-12**	1	2	3	
**Ethnicity (n)**				
**White**	13 (100%)	10 (91%)	11 (74%)	0.099
**Non-white**	0 (0%)	1 (9%)	4 (26%)	
**School lunch type (n)**				
**Packed Lunch**	5 (39%)	1 (9%)	0 (0%)	0.007
**School Provided Lunch**	0	7 (64%)	11 (73%)	
**Mix: School Provided or Packed**	5 (39%)	3 27%)	3 (20%)	
**At Home**	2 (15%)	0 (0%)	1 (7%)	
**Missing data**	1 (8%)	0 (0%)	0 (0%)	
**Household Income**				
**Less than £20,000**	1 (8%)	1 (9%)	0 (0%)	<0.001
**£20,000 - £40,000**	2 (15%)	1 (9%)	0 (0%)	
**£40,001 - £60,000**	8 (60%)	1 (9%)	1 (7%)	
**£60,001 - £80,000**	2 (15%)	5 (46%)	3 (20%)	
**Over £80,000**	0 (0%)	3 (27%)	11 (73%)	
**Highest Parental Qualification**				
**Level 1: one to four GCSE passes and any other GCSEs at other grades, or equivalent qualifications**	0	0	0	0.758
**Level 2: five or more GCSE passes or equivalent qualifications**	0	0	0	
**Apprenticeships**	0	0	0	
**Level 3: two or more A Levels or equivalent qualifications**	0	1 (9%)	1 (7%)	
**Level 4 or above: Higher National Certificate, Higher National Diploma, Bachelor’s degree, or post-graduate qualifications**	12 (92%)	8 (73%)	12 (80%)	
**Other qualifications, of unknown level**	1 (8%)	2 (18%)	2 (13%)	

* P-values represent comparisons of demographic characteristics between dietary groups (vegan, vegetarian and omnivore). Continuous variables were analysed using one-way Analysis of Variance (ANOVA) and categorical variables using chi-squared tests. Statistical significance was defined as p < 0.05.

** Although study recruitment was aimed at those aged 2–12, no 12-year-olds were recruited.

Most demographic characteristics did not differ significantly between dietary groups. Mean age was comparable across groups (p = 0.870), and there was no significant difference in sex distribution, although the majority of participants in each group were male. Ethnicity distribution was also similar between groups, with most participants identifying as white.

No significant differences were observed for index of multiple deprivation, parental education level, or marital status (all p > 0.05). Although overall differences in parental education level were not statistically significant, most parents were educated to degree level or higher (omnivores 100%, vegetarians 91%, vegans 93%).

Household income differed significantly between dietary patterns (p < 0.001), with omnivores reporting higher income than vegans in post-hoc comparisons. School lunch type also differed significantly between groups (p < 0.05), although post-hoc comparisons did not identify significant pairwise differences.

Supplement use varied markedly by dietary pattern and was significantly associated with diet type (p < 0.001). Supplement use was substantially higher among vegetarians and vegans compared with omnivores, with no significant difference between the two plant-based groups.

### Dietary intakes

Table 2 presents the percentage of nutrient requirements met across dietary groups, adjusted for age and sex, with and without supplementation.

**Table 2:** Mean percentage of UK Dietary Reference Values met by dietary pattern, with and without supplements, adjusted for age and sex (standard deviations shown in brackets).

**Nutrients		Calories	Total fat	Saturated fat	Protein	Carbs	Fibre	Free sugar	Vitamin B12	Vitamin A
**Diet**										
**Omnivore**	**No supplements**	99% (19%)	89% (19%)	103% (21%)	264% (65%)	97% (24%)	93% (23%)	110% (104%)	543% (320%)	113% (58%)
	**With supplements**	99% (19%)	89% (19%)	103% (21%)	264% (65%)	97% (24%)	93% (23%)	116% (114%)	557% (350%)	121% (56%)
**Vegetarian**	**No supplements**	89% (20%)	83% (23%)	93% (36%)	204% (67%)	91% (19%)	104% (25%)	82% (58%)	374% (373%)	149% (66%)
	**With supplements**	89% (20%)	83% (23%)	93% (36%)	204% (67%)	91% (19%)	104% (25%)	82% (58%)	419% (425%)	191% (73%)
**Vegan**	**No supplements**	98% (26%)	76% (22%)	54% (9%)	224% (55%)	104% (34%)	151% (42%)	50% (41%)	185% (55%)	171% (120%)
	**With supplements**	98% (26%)	76% (22%)	54% (9%)	224% (55%)	104% (34%)	151% (42%)	50% (41%)	207% (121%)	218% (131%)
*P-Value	**No supplements**	0.471	0.467	0.001	0.079	0.459	<0.001	0.124	0.008	0.210
	**With supplements**	0.471	0.467	0.001	0.079	0.459	<0.001	0.124	0.024	0.024
**Nutrients		**Vitamin C**	**Vitamin D**	**Folate**	**Iodine**	**Iron**	**Calcium**	**Zinc**	**Sodium**	
**Diet**										
**Omnivore**	**No supplements**	182% (79%)	20% (11%)	162% (53%)	115% (70%)	97% (30%)	126% (54%)	81% (20%)	103% (31%)	
	**With supplements**	194% (77%)	33% (37%)	170% (72%)	115% (70%)	101% (32%)	126% (54%)	85% (27%)	103% (31%)	
**Vegetarian**	**No supplements**	138% (71%)	13% (6%)	157% (48%)	81% (102%)	98% (58%)	109% (84%)	76% (30%)	103% (48%)	
	**With supplements**	230% (117%)	45% (34%)	236% (125%)	118% (99%)	131% (89%)	109% (84%)	107% (65%)	103% (48%)	
**Vegan**	**No supplements**	268% (176%)	25% (13%)	221% (95%)	40% (37%)	116% (41%)	117% (43%)	87% (44%)	103% (48%)	
	**With supplements**	327% (188%)	105% (36%)	267% (117%)	84% (51%)	122% (42%)	117% (43%)	97% (42%)	103% (48%)	
*P-Value	**No supplements**	0.032	0.034	0.042	0.035	0.478	0.780	0.713	0.255	
	**With supplements**	0.038	<0.001	0.054	0.459	0.401	0.780	0.427	0.255	

* P-values represent comparisons of mean percentage of nutrient recommendations met between dietary groups (omnivore, vegetarian and vegan). Differences were assessed using one-way Analysis of Variance (ANOVA). Statistical significance was set at p < 0.05.

** Percentages represent intake relative to UK Dietary Reference Values (DRVs). For micronutrients, Reference Nutrient Intake (RNI) values were used; for energy, Estimated Average Requirement (EAR) values were used; and for macronutrients, free sugars and fibre, age- and sex-specific recommended intake values from UK government dietary guidance were applied.

Overall, no dietary group met all nutrient reference values. Differences between dietary patterns were most evident for fibre, saturated fat, and several micronutrients.

PB diets were characterised by higher fibre intake and lower saturated fat intake compared with omnivorous diets. Fibre intake exceeded recommended levels in PB diets but remained below recommendations in omnivores. In contrast, saturated fat intake exceeded recommended levels in omnivores but not in vegetarian or vegan groups.

When supplements were included, statistically significant differences between dietary groups were observed for saturated fat, fibre, and vitamins A, C, B12 and D (all p < 0.05). Post-hoc analyses indicated that these differences were primarily between vegans and omnivores. Vegans had higher intakes of fibre, vitamin A, vitamin C and folate, and lower intakes of vitamin B12 compared with omnivores. Differences in saturated fat, fibre and vitamin D were also observed between vegans and vegetarians, with vegans consuming less saturated fat and more fibre and vitamin D.

When supplements were excluded, significant differences remained for saturated fat, fibre, vitamins B12, C and D, iodine, and folate (all p < 0.05). In this analysis, vegans had lower intakes of iodine and vitamin B12, but higher intakes of folate compared with omnivores. Differences between vegans and vegetarians were observed for vitamins C and D only, with higher intakes in vegans.

Supplement use had a clear impact on nutrient adequacy. Vitamin D intake was below recommended levels across all dietary groups when supplements were excluded, but only reached recommended levels in vegans when supplements were included. Iodine and zinc intakes also decreased without supplementation across all groups, with vegans remaining below recommended levels even when supplements were included.

Across all dietary patterns, protein intake exceeded recommended levels, while energy and total fat intakes were below recommendations.

**Fig 1:**
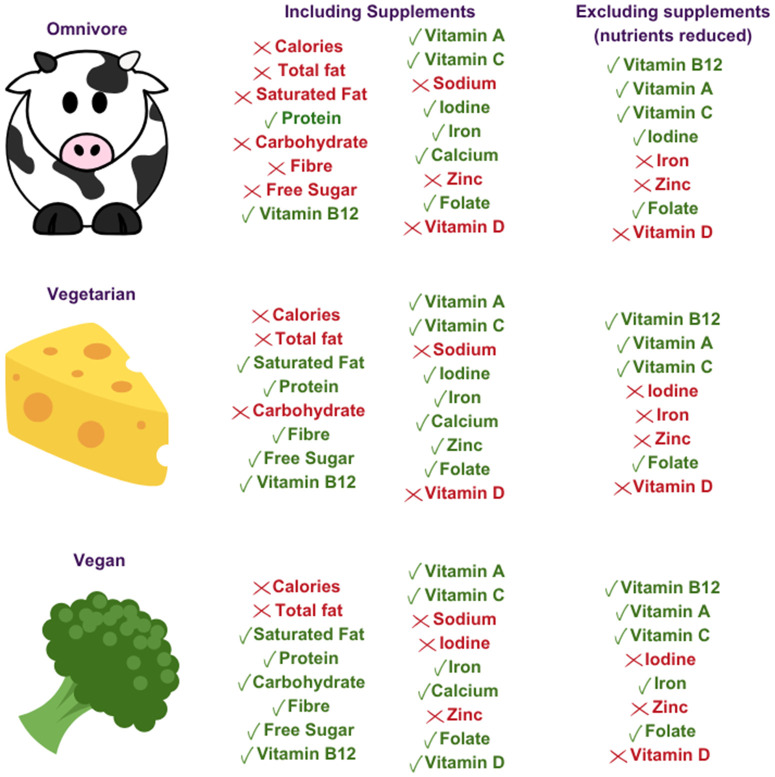
Visual summary of nutrient outcomes across omnivore, vegetarian and vegan diets in children aged 2–12 years, with and without supplements.

To aid interpretation of nutrient outcomes across dietary patterns, Fig 1 provides a visual summary of key nutrients for omnivore, vegetarian and vegan diets, both including and excluding supplements. Green ticks indicate nutrients consumed at a favourable level, while red crosses highlight nutrients where results suggest potential concern.

Table 3 presents the percentage contribution of macronutrients to total energy intake across dietary groups.

**Table 3:** Mean nutrient percentage of energy intake by macronutrients per day by dietary pattern* (standard deviation shown in brackets).

Diet	Omnivore	Vegetarian	Vegan	**P-Value
***Nutrients				
**% of energy from carbohydrate**	51.8 (4.97	54.8 (4.07)	56.4 (7.37)	0.124
**% of energy from protein**	15.4 (2.16)	12.9 (2.03)	13.2 (2.11)	0.006
**% of energy from fat**	32.8 (4.20)	32.3 (3.64)	30.5 (7.45)	0.504
**% of energy from saturated fat**	12.4 (2.96)	11.2 (3.68)	7.7 (2.25)	<0.001

* These values are the same when supplements are both included and excluded, as macronutrients remained unchanged.

** P-values represent comparisons of the mean percentage of energy intake from macronutrients between dietary groups (omnivore, vegetarian and vegan). Differences were analysed using one-way Analysis of Variance (ANOVA). Statistical significance was set at p < 0.05.

*** Recommended percentage of energy intake: carbohydrate 50%, protein 15%, total fat ≤ 35%, saturated fat ≤ 11% (UK dietary guidelines).

All dietary patterns exceeded the recommended contribution from carbohydrates (50% of total energy), with no significant differences observed between groups (p = 0.124). Total fat intake was below the recommended 35% of energy intake in all groups, with no significant differences between dietary patterns (p = 0.504).

Protein intake as a percentage of total energy differed significantly between groups (p = 0.006). Omnivores were the only group to meet the recommended 15% contribution from protein, while vegetarians and vegans fell slightly below this level.

For saturated fat, omnivores and vegetarians exceeded the recommended limit of 11% of total energy intake, whereas vegans remained below this threshold. This difference was statistically significant (p < 0.001), with lower saturated fat intake observed in vegans compared with both omnivores and vegetarians.

Table 4 presents the most frequently reported food contributors to key nutrients across dietary patterns. Among omnivores, animal-based foods were the most common contributors to several nutrients, with dairy products appearing as a key source of vitamin B12, iodine, calcium and protein. Meat products, including beef, pork and chicken, were also common contributors to nutrients such as protein and vitamin B12.

**Table 4:** Primary food sources of key nutrients across omnivore, vegetarian and vegan diets.

	Omnivore	Vegetarian	Vegan
**B12**	Dairy	Dairy	Dairy alternatives
	Beef	Eggs	Nutritional Yeast
	Pork	Nutritional Yeast	
**Iron**	Breakfast cereal	Breakfast cereal	Tofu
	Peas	Bread	Breakfast cereal
		Baked beans	Dairy alternatives
**Iodine**	Dairy	Supplement	Dairy alternative
	Eggs	Dairy	Supplement
		Eggs	
**Vitamin D**	Breakfast cereal	Supplement	Supplement
	Dairy	Eggs	Dairy alternatives
	Pork	Dairy alternatives	Breakfast cereal
**Calcium**	Dairy	Dairy	Dairy alternatives
	Breakfast cereal	Bread	Bread
		Dairy alternatives	
**Protein**	Dairy	Dairy	Bread
	Chicken	Meat alternatives	Dairy alternatives
	Beef	Eggs	Tofu

Fortified breakfast cereals appeared as an important contributor across all dietary groups, particularly for iron and vitamin D. Among vegetarian participants, dairy and eggs were common contributors to several nutrients, although supplements were also frequently reported as a source of iodine and vitamin D.

Among vegan participants, fortified foods were prominent contributors to several nutrients. Fortified dairy alternatives were commonly reported sources of calcium, vitamin B12, vitamin D and iodine, while nutritional yeast contributed to vitamin B12 intake. Tofu and bread also appeared as common contributors to iron and protein. Supplements were reported as contributors to vitamin D and iodine in some cases.

### Environmental analysis

Table 5 presents mean daily carbon footprints (CO_2_e) across dietary groups and by Eatwell Guide food categories.

**Table 5:** Comparison of mean daily carbon footprints (kgCO₂e) across dietary groups and for each Eatwell Guide section, with significance values.

Diet type	Omnivore	Vegetarian	Vegan	P-Value
**Mean daily** **(St.Dev)**	2.53 (1.04)	1.70 (0.61)	1.36 (0.43)	<0.001
**Carbohydrate (St.Dev)**	0.283 (0.119)	0.292 (0.195)	0.255 (0.115)	0.774
**Protein (St.Dev)**	1.174 (0.894)	0.120 (0.095)	0.214 (0.154)	<0.001
**Dairy (St.Dev)**	0.434 (0.249)	0.357 (0.390)	0.165 (0.100)	0.033
**Fruit and Vegetables (St.Dev)**	0.193 (0.087)	0.207 (0.158)	0.320 (0.223)	0.101
**Fats (St.Dev)**	0.033 (0.031)	0.024 (0.017)	0.017 (0.040)	0.400
**HFSS (St.Dev)**	0.397 (0.248)	0.686 (0.584)	0.373 (0.278)	0.098
**Other (St.Dev)**	0.014 (0.026)	0.009 (0.012)	0.021 (0.032)	0.524

Overall, mean daily carbon footprints differed significantly between dietary patterns (p < 0.001). Omnivorous diets had the highest carbon footprints, followed by vegetarian diets, with vegan diets having the lowest. Post-hoc analyses indicated that omnivore diets had significantly higher carbon footprints than both vegetarian and vegan diets, while no significant difference was observed between vegetarian and vegan diets.

Analysis by Eatwell Guide category identified significant differences for protein and dairy (both p < 0.05). For protein, carbon footprints were highest in omnivores and significantly lower in both vegetarian and vegan groups, with no significant difference between the two plant-based groups. For dairy, carbon footprints were also highest in omnivores and lowest in vegans, with a significant difference observed between these groups, but not between vegetarians and the other dietary patterns.

No significant differences were observed between dietary groups for carbohydrates, fruit and vegetables, fats, HFSS foods, or the ‘other’ category (all p > 0.05).

Although vegans had the highest mean carbon footprints for fruit and vegetables, ‘other’ omnivores for fats, and vegetarians for HFSS foods and carbohydrates, these differences did not reach statistical significance at the 5% level.

## Discussion

This cross-sectional study assessed the nutritional adequacy and environmental impact of omnivorous, vegetarian, and vegan diets among UK children aged 2–12 years. While no dietary pattern met all nutrient reference values, PB diets were associated with lower carbon footprints and more favourable intakes of fibre, saturated fat, and free sugars compared with omnivorous diets. However, vegan diets did have some important limitations, particularly regarding low iodine intake, even with supplementation.

Our study finds that children following omnivorous diets exceeded recommended intakes for saturated fat and free sugars while failing to meet the recommended intake for fibre, which mirrors the recent NDNS findings [6], In contrast, vegetarian and vegan diets were characterised by higher fibre and lower saturated fat and free sugar intakes, aligning with previous studies [8,10,24]. These differences are likely to have important health implications, as higher fibre intake is associated with improved gut health and reduced long-term risk of conditions such as colorectal cancer [25], while high consumption of free sugars is linked to obesity, dental disease and type 2 diabetes [26,27]. Together, these findings suggest that PB diets may offer advantages in terms of overall dietary quality in children.

Protein intake exceeded recommended levels across all dietary groups, indicating that PB diets can meet protein requirements in children. However, when expressed as a proportion of total energy intake, vegetarians and vegans fell slightly below recommended levels. While total protein intake appears sufficient, this study did not assess amino acid profiles, and previous research suggests that plant-based diets may be lower in certain essential amino acids [28,29], highlighting an area for further investigation in children.

Several micronutrients differed between dietary patterns. Vitamin B12 intake was sufficient across all groups, including vegans, even in the absence of supplementation. This contrasts with some international findings [8] and likely reflects the widespread B12 fortification of PB foods in the UK [30]. However, not all PB alternatives are nutritionally equivalent; recent evidence suggests that many plant milks do not provide a like-for-like replacement for cow’s milk in terms of micronutrient content [31], emphasising the importance of appropriate product selection and fortification.

In contrast, iodine intake remained inadequate in vegan children, even when supplements were included, consistent with previous research highlighting iodine as a nutrient of concern in PB diets [32]. This may reflect limited iodine fortification in PB alternatives compared with dairy products. Zinc intake was also below recommended levels in most groups without supplementation, with only vegetarians meeting requirements when supplements were included. These findings differ from NDNS data, which suggests most children meet zinc requirements [6], and may reflect differences in dietary composition or potential selection bias in the small sample size.

Vitamin D intake was below recommended levels across all dietary patterns without supplementation, consistent with national data [6]. Only vegan children met recommended levels when supplements were included, likely reflecting higher supplement use in this group. This reinforces existing public health guidance recommending vitamin D supplementation in UK children, particularly during months with limited sunlight exposure.

Energy and total fat intakes were below recommended levels across all dietary groups. While this could suggest insufficient intake, it is more likely due to under-reporting, a well-documented limitation in dietary assessment studies and consistent with NDNS findings [6].

From an environmental perspective, omnivorous diets had significantly higher carbon footprints than both vegetarian and vegan diets, consistent with previous research in adult populations [13,14]. These differences were primarily driven by higher GHG emissions from animal-based protein and dairy products, which are among the most emissions-intensive food groups [11,12], and supports recommendations for more plant-based dietary patterns such as the EAT-Lancet planetary health diet [3].

Evidence on the environmental impact of children’s diets remains limited, particularly in the UK. The findings of this study are consistent with emerging international research in children, which has reported higher carbon footprints in diets high in animal products [33,34], although these studies emphasise the need to consider nutritional adequacy alongside environmental sustainability. In the present study, differences in carbon footprints were primarily driven by animal-based protein and dairy, with no significant differences observed across other food groups. This highlights the importance of reducing these food groups as a key strategy for lowering dietary greenhouse gas emissions and carbon footprints.

However, an important consideration is the balance between environmental sustainability and nutritional adequacy. Low-carbon foods are not always nutritionally equivalent replacements, and poorly fortified PB alternatives may increase the risk of micronutrient inadequacy, particularly for nutrients such as iodine [35]. These findings emphasise the need to evaluate both nutritional and environmental outcomes when assessing sustainable diets in children.

This study provides novel insights into both the nutritional adequacy and carbon footprints of PB diets in UK children. Strengths include the use of three-day weighed food diaries, which provide greater accuracy than commonly used dietary assessment methods, and the inclusion of both nutritional and environmental analyses. The dual analysis of nutrient intake with and without supplementation also offers a more comprehensive assessment of dietary adequacy.

However, several limitations should be considered. The small sample size and use of convenience sampling limit the generalisability of the findings. As well as this, the study aimed to include children aged 2–12 years, however, no 12-year-olds were recruited, resulting in a slightly narrower age range than intended, which may limit representation of older children within the sample. Dietary intake was assessed over three days and may not reflect usual intake, with under-reporting likely. The study did not assess biochemical markers of nutrient status or amino acid profiles, limiting conclusions regarding nutrient bioavailability and protein quality. Additionally, environmental impacts were assessed using GHE emissions and carbon footprints only and did not include other factors such as water use or packaging.

Future research should include larger, more representative samples and assess long-term health outcomes of plant-based diets in children. Studies incorporating biochemical measures of nutrient status and amino acid profiles would provide further insight into nutritional adequacy. Further research is also needed to assess the accessibility and affordability of plant-based diets to ensure equitable dietary recommendations. These findings highlight the importance of dietary guidance that supports nutritional adequacy and long-term health outcomes in children, while also promoting planetary health.

## Conclusion

This study is the first to assess both the nutritional adequacy and carbon footprints of omnivorous, vegetarian, and vegan diets in UK children aged 2–12 years. While no dietary pattern met all nutrient recommendations, PB diets were associated with higher intakes of fibre, folate, and vitamins A and C, and lower intakes of saturated fat and free sugars compared with omnivorous diets. However, nutrients including iodine, vitamin D, and zinc remain areas of concern, particularly in the absence of appropriate supplementation.

From an environmental perspective, omnivorous diets had significantly higher carbon footprints than vegetarian and vegan diets, reinforcing the environmental benefits of reducing animal-based food consumption.

These findings highlight the importance of dietary guidance that supports nutritional adequacy and long-term health outcomes in children, while also promoting planetary health.

## Supporting information

S1 File Participant full demographic information. (DOCX)
