# Supplementary material for: Nutritional availability and carbon footprints of omnivorous, vegetarian and vegan diets: A cross-sectional analysis of dietary data for UK children aged 2–12
Source: PLoS One. 2026 Jun 18;21(6):e0342629. doi: 10.1371/journal.pone.0342629 (PMC13278396; doi:10.1371/journal.pone.0342629)
Supplement: S1 File — (DOCX) [file pone.0342629.s001.docx]

S1 Table

| S1 table: Demographic and dietary characteristics of study participants included in quantitative dietary analysis (children aged 2–12 years) | | | | |
| --- | --- | --- | --- | --- |
| Diet Pattern (overall % of participants) | Vegan (33%) | Vegetarian (28%) | Omnivore (39%) | P-Value |
| Study Size 39 | 13 | 11 | 15 | n/a |
| Supplement use |  | | | |
| Yes | 12 (92%) | 9 (82%) | 2 (13%) | <0.001 |
| No | 1(8%) | 2 (18%) | 13 (87%) |  |
| Sex |  |  |  |  |
| Female | 4 (31%) | 4 (36%) | 6 (40%) | 0.878 |
| Male | 8 (69%) | 7 (64%) | 9 (60%) |  |
| Age (years) | Mean= 5.2 (std.dev=2.68) | Mean= 5.73 (std.dev=2.72) | Mean= 5.23 (std.dev=2.80) |  |
| 2 | 3 (23%) | 1 (9%) | 2 (13%) | 0.870 |
| 3 | 2 (15%) | 2 (18%) | 4 (27%) |  |
| 4 | 0 (0%) | 1 (9%) | 1 (7%) |  |
| 5 | 2 (15%) | 1 (9%) | 2 (13%) |  |
| 6 | 2 (15%) | 3 (27%) | 1 (7%) |  |
| 7 | 1 (8%) | 0 (0%) | 2 (13%) |  |
| 8 | 2 (15%) | 1 (9%) | 0 (0%) |  |
| 9 | 0 (0%) | 0 (0%) | 2 (13%) |  |
| 10 | 0 (0%) | 2 (18%) | 1 (7%) |  |
| 11 | 1 (8%) | 0 (0%) | 0 (0%) |  |
| Ethnicity (n) |  |  |  |  |
| White | 13 (100%) | 10 (91%) | 11 (74%) | 0.099 |
| Non-white | 0 (0%) | 1 (9%) | 4 (26%) |  |
| School Type (n) |  |  |  |  |
| State School (non-selective) | 5 (39%) | 8 (73%) | 8 (53%) | 0.200 |
| Home Schooled/Stay at home | 2 (15%) | 0 (0%) | 1 (7%) |  |
| Flexi school: Part home schooled/part school | 2 (15%) | 0 (0%) | 0 (0%) |  |
| Private School | 0 | 0 (0%) | 1 (7%) |  |
| Nursery | 3 (23%) | 3 (27%) | 5 (33%) |  |
| Childminder | 1 (8%) | 0 (0%) | 0 (0%) |  |
| School lunch type (n) |  |  |  |  |
| Packed Lunch | 5 (39%) | 1 (9%) | 0 (0%) | 0.007 |
| School Provided Lunch | 0 | 7 (64%) | 11 (73%) |  |
| Mix: School Provided or Packed | 5 (39%) | 3 27%) | 3 (20%) |  |
| At Home | 2 (15%) | 0 (0%) | 1 (7%) |  |
| Missing data | 1 (8%) | 0 (0%) | 0 (0%) |  |
| Index of Multiple Deprivation |  |  |  |  |
| Unavailable | 4 (31%) | 2 (18%) | 0 (0%) | 0.052 |
| 1 | 0 (0%) | 0 (0%) | 0 (0%) |  |
| 2 | 0 (0%) | 0 (0%) | 0 (0%) |  |
| 3 | 1 (8%) | 0 (0%) | 0 (0%) |  |
| 4 | 2 (15%) | 3 (27%) | 1 (7%) |  |
| 5 | 0 (0%) | 1 (9%) | 4 (27%) |  |
| 6 | 2 (15%) | 1 (9%) | 1 (7%) |  |
| 7 | 1 (8%) | 0 (0%) | 0 (0%) |  |
| 8 | 1 (8%) | 1 (9%) | 6 (40%) |  |
| 9 | 1 (8%) | 3 (27%) | 0 (0%) |  |
| 10 | 1 (8%) | 0 (0%) | 3 (20%) |  |
| Household Income |  |  |  |  |
| Less than £20,000 | 1 (8%) | 1 (9%) | 0 (0%) | <0.001 |
| £20,000 - £40,000 | 2 (15%) | 1 (9%) | 0 (0%) |  |
| £40,001 - £60,000 | 8 (60%) | 1 (9%) | 1 (7%) |  |
| £60,001 - £80,000 | 2 (15%) | 5 (46%) | 3 (20%) |  |
| Over £80,000 | 0 (0%) | 3 (27%) | 11 (73%) |  |
| Parental marital Status |  |  |  |  |
| Married/same sex civil partnership | 9 (69%) | 7 (64%) | 15 (100%) | 0.082 |
| Cohabiting | 4 (31%) | 3 (27%) | 0 (0%) |  |
| Divorced/separated | 0 (0%) | 1 (9%) | 0 (0%) |  |
| Highest Parental Qualification |  |  |  |  |
| No qualifications |  |  |  | 0.758 |
| Level 1: one to four GCSE passes (grade A* to C or grade 4 and above) and any other GCSEs at other grades, or equivalent qualifications | 0 | 0 | 0 |  |
| Level 2: five or more GCSE passes (grade A* to C or grade 4 and above) or equivalent qualifications | 0 | 0 | 0 |  |
| Apprenticeships | 0 | 0 | 0 |  |
| Level 3: two or more A Levels or equivalent qualifications | 0 | 1 (9%) | 1 (7%) |  |
| Level 4 or above: Higher National Certificate, Higher National Diploma, Bachelor's degree, or post-graduate qualifications | 12 (92%) | 8 (73%) | 12 (80%) |  |
| Other qualifications, of unknown level | 1 (8%) | 2 (18%) | 2 (13%) |  |
